# Do Haematophagous Bugs Assess Skin Surface Temperature to Detect Blood Vessels?

**DOI:** 10.1371/journal.pone.0000932

**Published:** 2007-09-26

**Authors:** Raquel A. Ferreira, Claudio R. Lazzari, Marcelo G. Lorenzo, Marcos H. Pereira

**Affiliations:** 1 Laboratório de Triatomíneos e Epidemiologia da Doença de Chagas, Centro de Pesquisas René Rachou, Belo Horizonte, Brazil; 2 Institut de Recherche sur la Biologie de l'Insecte, UMR CNRS 6035, Université François Rabelais, Tours, France; 3 Laboratório de Fisiologia de Insetos Hematófagos, Universidade Federal de Minas Gerais, Belo Horizonte, Brazil; Freie Universitaet Berlin, Germany

## Abstract

**Background:**

It is known that some blood-sucking insects have the ability to reach vessels under the host skin with their mouthparts to feed blood from inside them. However, the process by which they locate these vessels remains largely unknown. Less than 5% of the skin is occupied by blood vessels and thus, it is not likely that insects rely on a “random search strategy”, since it would increase the probability of being killed by their hosts. Indeed, heterogeneities along the skin surface might offer exploitable information for guiding insect's bites.

**Methodology/Principal Findings:**

We tested whether the bug *Rhodnius prolixus* can evaluate temperature discontinuities along the body surface in order to locate vessels before piercing the host skin. When placed over a rabbit ear, the bug's first bites were mostly directed towards the main vessels. When insects were confronted to artificial linear heat sources presenting a temperature gradient against the background, most bites were directly addressed to the warmer linear source, notwithstanding the temperature of both, the source and the background. Finally, tests performed using uni- and bilaterally antennectomized insects revealed that the bilateral integration of thermal inputs from both antennae is necessary for precisely directing bites.

**Conclusions/Significance:**

*R. prolixus* may be able to exploit the temperature differences observed over the skin surface to locate blood vessles. Bugs bite the warmest targets regardless of the target/background temperatures, suggesting that they do not bite choosing a preferred temperature, but select temperature discontinuities along the skin. This strategy seems to be an efficient one for finding blood vessels within a wide temperature range, allowing finding them on different hosts, as well as on different areas of the host body. Our study also adds new insight about the use of antennal thermal inputs by blood sucking bugs.

## Introduction

Solenophagous insects such as mosquitoes, sucking lice and kissing bugs need to pierce the skin of their hosts in order to reach the interior of blood-vessels with their mouthparts [1]. How do they perform this has not been analysed in detail in any species, despite the relevance of vessel localisation for successful haematophagy and even for parasite transmission.

*Rhodnius prolixus*, in addition of being a classical model for insect physiology studies, is a major vector of Chagas' disease, one of the main public health problems in Latin America [2]–[4]. Endemic to 21 countries, the disease affects approximately 16–18 million people, while other 120 million are exposed to risk of transmission [5]. In Venezuela, Colombia and some parts of Central America, *R. prolixus* Stål (1859) is the main vector of the disease [6].

The heat emitted by warm blooded animals is the main cue used by haematophagous bugs to locate a host at short distances [7]–[9]. Indeed, heat is the only stimulus both, necessary and sufficient to trigger biting in these insects [10]. For the detection of heat sources, they use thermoreceptors mainly localized on their antennae [9]–[10]. These sense organs allow them to discriminate between heat sources of different temperature and determine their position in space [7]–[10]. When insects are in close proximity of a warm object, they extend their proboscis to reach the target and bite it. At that phase, bilateral inputs coming from both antennae are integrated to precisely locate the goal [9]–[10]. The role of heat as a cue for finding food by triatomine bugs has been studied in relation to host location, but its role during biting remains relatively unknown. In this sense, there is one study [11] showing that bugs are able to bite cold surfaces and feed on blood kept at a temperature of just a few degrees Celsius, provided that their antennae are stimulated by heat. However, the role of heat in vessel location has not been investigated so far.

*R. prolixus,* a solenophagous bug, obtains food directly from venules and arterioles [12]. After piercing the host skin, the insect moves the mouthparts under the epidermis until it reaches a blood vessel and penetrates it. This probing activity represents only 6% of the total contact time with the host during feeding [13]. It is a critical phase, because the insertion of the mouthparts and their movements under the skin cause tissue damage and the release of chemicals that can trigger nerve stimulation, haemostasis and inflammatory reactions [14]. Therefore, finding blood vessels quickly and without disturbing the host by piercing its skin repetitively might have been a key feature for the adaptation of these bugs to haematophagy.

In the present investigation, we show that *R. prolixus* can locate a blood vessel before contacting the host skin with its proboscis. Moreover, we provide evidence supporting the role of the thermal sense and the antennae in guiding this behaviour.

## Results and Discussion

### Targeting bites

When we confronted the bugs with the ear of a rabbit, we observed that 77% (24/31) of their first bites (i.e., the very first contact of the proboscis of each insect with the skin) were oriented towards a major vessel or up to 2 mm away from it. We conclude that this distribution of bites indicates that these insects exploit sensory cues associated to blood vessels to detect them before contacting the host skin with their proboscis. Vessels revealed to be the warmest areas on the rabbit's ear, as the temperature over them was 32.5±0.5°C and gradually decreased to 31±0.4°C up to 10 mm away.

Subsequently, we evaluated whether heat is used as a cue for vessel location by performing two experiments in which linearly-shaped heat sources were presented over lower temperature backgrounds, i.e., bugs were confronted to a heated wire presented over a metal plate that acted as background. Both the wire and the plate could have their temperatures controlled independently.

**Figure 1:**
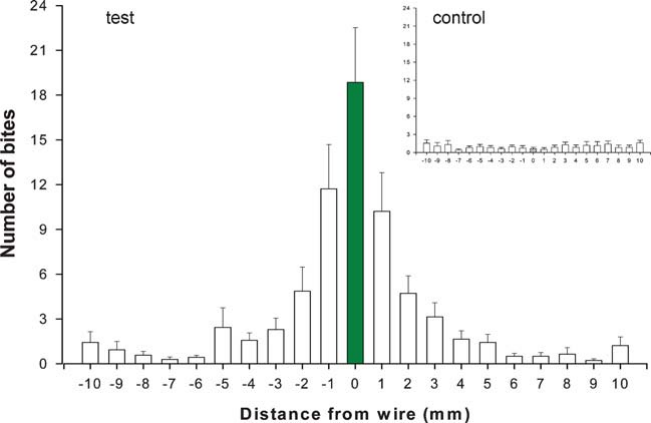
Mean distance of the sites of bites in relation to the position of the heated wire. Test: the wire was presented at 36°C and the background at 33°C. Control: both, the wire and the background were kept at 33°C. Each bar represents the mean value plus the standard error of the number of bites perfomed over the wire (green bar) and at different distances from it by 14 insects along a period of 10 min after the first bite occurred.

In the first experimental series, we kept the linearly shaped source at 36°C over a background at 33°C. These assays revealed that bites were aimed either to the linear heat source or to its proximity (Figure 1), with a mean distance of 1.6±0.6 mm between the spot contacted by the proboscis and the linear source. Control assays keeping the linear heat source at the same temperature as the background (i.e., 33°C), revealed a quite homogeneous distribution of bites, with mean distance of 5.4±2.2 mm between the linear source and the spot contacted by the proboscis of bugs (Figure. 1). This mean distance of the bites to the linear source resulted significantly larger than that observed in the first experimental series (Mann Whitney, p = 0.0003).

### Absolute vs. relative temperature

In the second experimental series, the linearly shaped heat source was kept at 39°C and the background at 36°C. Again, most bites where directed towards the linear heat source. Consistently, we did not observe such a pattern when the linear source was presented at the same temperature as the background (i.e., 36°C). The mean distance between the spot contacted by the proboscis and the warmer linear heat source was 2.6±1.1 mm. In the control assays that presented both, linear source and background at 36°C, a significantly larger distance of 6.6±1.3 mm was observed (Mann Whitney, p = 0.0022).

Previous work on triatomines revealed that these insects prefer warm objects within a certain temperature range that can attract bugs and evoke proboscis extension [7]–[10]. Our findings go further, indicating that bugs do not select absolute temperatures when choosing a place to bite, but thermal contrasts between their targets and the background. This was revealed by the experiments in which insects were confronted with backgrounds at 33 or 36°C that presented 3°C warmer linear heat sources over them. Different hosts present diverse surface temperatures, e.g., a bird may have a higher body temperature than a mammal. Moreover, different parts of the body of a host have distinct surface temperatures. To respond to thermal differences and not to absolute temperatures would allow bugs to find vessels over a wide temperature range, because vessels are always warmer than the surrounding tissues.

**Figure 2:**
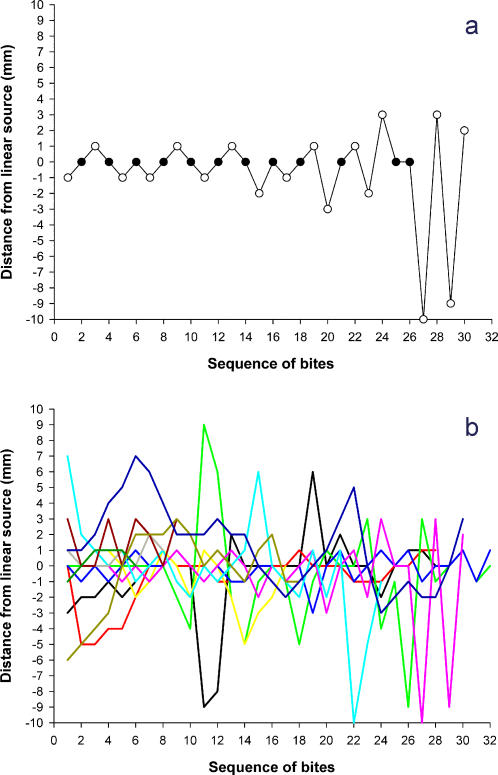
Sample records of the distance from an initial sequence of bites to the linear thermal source, performed by insects in an assay in which the linear source was 3°C warmer than the background. a, Sample record corresponding to a single insect; black circles represent direct contacts with the heated wire. b, The distribution of bites performed by several insects, showing the location of the spots contacted in consecutive biting attempts. Each sequence represents approximately one minute.

On the other hand, our experiments revealed that insects were able to directly contact the warmer wire since the first bites (Figure 2a). Thus, insects should be able to locate the target even before touching the surface with their proboscis. Despite no meal was found by the insects, they did not resign, but continued biting the heat source. However, they alternated bites on the wire and at some distance at each side of the linear source showing an apparently zigzagging pattern of sequential bites (Fig. 2b). We suggest that the role of this biting pattern may be to increase the chance of contacting the real blood source by repetitive biting being performed uninterruptedly as far as an expected subsequent stimulus is not detected by their proboscis after physical contact, e.g., skin texture, skin chemistry or vessel properties like periodic vibrations.

### The role of the antennae

**Figure 3:**
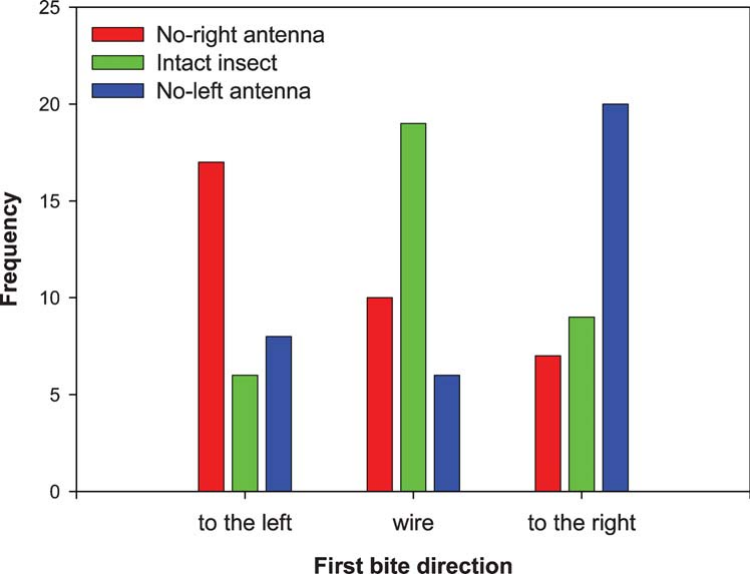
Distribution of first bites (i.e., the first contact of each insect with the source) performed by intact (n = 34) and unilaterally antennectomized bugs (n = 34 left+34 right) in relation to the position of the heated wire. Only intact insects directed their bites correctly towards the heat source, whereas bugs lacking one antenna missed the goal and addressed their bites in the direction of the remaining antenna. Fully antennectomized insects (n = 20) did not respond at all and for this reason they have not been included in the plot.

In a third series of experiments, we tested the ability of uni- and bilaterally antennectomized insects to locate the linearly shaped heat source. Results have confirmed that, as it happens during the approach of the insect to a thermal source [9], [10], the bilateral integration of antennal inputs is needed to guide the insect's biting attempts (Figure 3). If one input is abolished by unilateral antennectomy, the insects miss the target, addressing their bites mostly to the zone located in the side of the remaining antena. This result strongly supports that the thermal receptors located on the antennae are responsible for blood vessel detection. In addition, this experiment reveals that a thermo-tropotaxis mechanism is involved in guiding proboscis extension.

Different thermoreceptors than those known from the antenna would play a secondary role in guiding the proboscis. In fact, bilaterally antennectomized insects revealed to be unable to locate the heat source or even extend their proboscis [9], [15]. This is quite surprising, because in other species of triatomines thermosensitive coeloconic sensilla are present on the antennae, but also on other regions of the body and, as a consequence, antennectomy does not abolish thermal sensitivity [15], [16]. Three possible explanations can be proposed for the loss of the capacity to locate a warm source resulting from antennectomy. First, antennectomy can affect the normal behaviour in such a way that bugs stop responding to stimuli of certain modalities. Second, thermoreceptors can be present on the proboscis, but only antennal ones would guide biting. Finally, it can be a characteristic of *R. prolixus* to lack thermoreceptors other than those of the antennae, or at least not to have them on their proboscis The first possibility is apparently supported by previous work that revealed that antennectomy deeply affects the normal behaviour of other bugs. They stop responding to warm objects by approaching them, and change to visually following moving objects or even extending their proboscis towards them [9]. Besides, even if they are still capable of synchronizing light or thermal environmental cycles, the expression of their daily activity cycles becomes altered [16]. Nevertheless, the lack of one antenna did not inhibit biting, since unilateral antennectomy induced the insects to perform the kind of systematic errors exactly expected from tropotactic integration of antennal inputs. This suggests that partial antennectomy does not inhibit thermodetection. The second option also seems less probable, since it is difficult to consider that the function of rostral thermoreceptors could be dissociated from biting and feeding. Regarding the third possibility, the existence of thermo-hygrosensitive coeloconic sensilla on the proboscis of *R. prolixus* has been proposed, but not conclusively shown up to date [17], [18]. To verify this, we searched for coeloconic sensilla using a scanning electron microscope (SEM) and no coeloconic thermo-hygroreceptors could be observed on the proboscis of *R. prolixus*. Furthermore, as indicated above, triatomine bugs are able to feed on blood at 3°C, provided that their antennae are conveniently stimulated by heat [11]. In that case, if thermal receptors were present on the proboscis, the cold temperature of the blood source would have constituted an aversive stimulus, tending to inhibit feeding.

The fact that intact bugs managed to finely determine the location of thermal sources and that unilaterally antennectomized bugs failed to aim their proboscis in a biased manner suggests that proboscis sensors, if any exist, are not involved in mediating this task. It is relevant to state that the proboscis is mostly retracted under the head of the bugs, while the antennae are mostly positioned forward. After a heat source is located at very short distance [9]–[10], bugs extend their proboscis in order to bite. This also suggests that the proboscis is not involved in the detection of heat sources. We propose here that the thermal stimuli used for vessel and heat source location are exclusively detected by thermal sensors situated in bugs' antennae. Moreover, this information must be integrated bilaterally in order to trigger the precise responses observed in intact bugs.

Even when thermoreceptors may exist outside the antennae of *R. prolixus*, they may be associated to functions other than targeting a heat source. The presence of different types of thermoreceptors associated to different kinds of thermosensitivity has been previously reported in insects. In a temperature gradient, *Drosophila melanogaster* flies loose their normal preference for 24°C when antennectomized, but continue avoiding temperatures above 31°C [19]. Besides, it has been suggested for the same species that antennal low-temperature thermosensors mediate spatial orientation processes and high-temperature thermosensors located outside the antennae are associated with spatial memories [20]. Thermal nociception is another form of thermosensitivity, which in both larvae and adults of *D. melanogaster* depends on the expression of a single gene, *painless* [21], [22].

### Searching for a vessel

An interesting aspect of the behaviour of triatomines confronted to thermal sources is that when an insect does not succeed to pierce the skin at a spot, it begins to bite on the proximities. This behaviour was first observed in *Triatoma infestans*, when stimulated by a warm metal plate associated to a container offering cold blood [11]. As no reward was obtained from the plate, bugs started biting nearby spots until they found the blood meal. A similar behaviour has been revealed here for *R. prolixus*. Our results revealed that that after performing several biting attempts on the linearly shaped heat source, the bugs started to bite apart from it. Thus, bugs do not limit their searches to the precise origin of their guiding stimuli, but extend them to nearby spots as an apparent strategy to increase their chances of contacting blood vessels in case their piercing attempts are unsuccessful.

This study adds new insight about how haematophagous insects locate vessels hidden under the skin. Heat appears as a main cue for aiding insects to perform this task. Even though other signals, as odours, humidity and vibrations cannot be excluded, heat alone seems to be sufficient. This is not surprising if we consider that these insects possess a highly developed thermal sense that allows them to detect and locate a host and that heat is the most important cue for host location in triatomine bugs, being indispensable to evoke biting.

As far as we know, this is the first evidence of the ability of a haematophagous insect to locate and subsequently probe the warmest sites on the host skin surface. We hypothesize that this capacity would improve the chances of finding a blood vessel, minimizing both the time consumed and the disturbance of the host. In addition, our work extends our comprehension about heat perception and the active use of heat cues by animals, a sensory capacity poorly understood.

The use of the thermal sense has been relative neglected in other haematophagous insects. Actually, most efforts on the study of host associated cues in vectors of human diseases have been addressed to chemical cues and olfactory orientation, particularly for mosquitoes. Provided that most blood-sucking insects have been submitted to similar selective pressures associated to food finding, we should not be surprised if future research shows that heat is more relevant than believed to date as a cue for food finding in mosquitoes, tse-tse flies and other hematophagous arthropods.

## Methods

### Insects

Fifth-instar *R. prolixus* larvae starved for 30–45 days after moulting were used throughout the experiments. The insects had their eyes covered with black acrylic paint in order to prevent the use of visual cues. For the final experiment, 3 groups of 34 insects each, where either left-, right- antennectomized or kept intact as control and 20 bugs were bilaterally antenectomized. The antennectomy of the experimental insects was performed 24 h before the onset of assays.

### Thermal measures on rabbit ears and thermal sources

The temperature on the skin surface over the vessel called *ramus intermedius* (diameter about 900 µm) and its surroundings were measured before every assay of the first experimental series (n = 31) using a contact thermometer (TES 1300, Taiwan, accuracy: 0.1°C). The values presented are the mean temperature differences between each vessel and the tissues located 1 cm away from it. The reported temperature for the thermal sources, i.e., wire or flat background, represents the values programmed by means of electronic thermostats (accuracy, 0.1°C). These temperatures were measured for verification before and after each trial.

### Thermal sources

The thermal source was an aluminium plate (flat background) and a nickel-chrome wire (linearly shaped thermal source). The nickel-chrome wire (300 µm thickness) was fastened on the aluminium plate and both were thermostatized at specific temperatures (accuracy: 1°C). In the first experimental series that used artificial thermal sources, the temperatures of the wire and the background were 36° and 33°C, respectively. In the corresponding control series, both thermal sources were presented at 33°C. In the second experimental series, the temperatures of the linear and the flat thermal sources were 39 and 36°C, respectively. In the control series for this experiment, both thermal sources were presented at 36°C.

### Experimental arenas

Each insect was placed into a receptacle located in one end of an acrylic rectangular box (24×16×8 cm) and released after 10 minutes. A square opening (2 cm wide) located on the substrate of the box, 10 cm away from the receptacle and in a central position of the box, allowed the insect to contact either the skin of the rabbit's ear or the artificial thermal sources. In the experiment presenting thermal sources to antennectomized insects, the arena was modified in order to allow the insects to reach the thermal source parallel to the heated wire. The new arena consisted of an acrylic corridor of 10×1 cm in which the heated wire was presented in a central position parallel to the arena's longitudinal axis. The new flat thermal source was identical to the previous one, but its area was diminished to 1 cm^2^ in order to restrict the access of the bugs to a direction parallel to the wire. In this manner, the stimulus was presented to the insects aligned with their longitudinal axis and, therefore, it was possible for us to assign their initial bites to the wire, the left or the right half of the flat source, in order to check if the distribution pattern observed in the experiments with intact insects was maintained.

### Data recording and analysis

A video camera sensitive to infrared (IR) light, was used to record their behaviour. The camera was mounted over the arena in a zenithal position relative to the thermal source, and the scene illuminated by a panel of IR emitting LEDs (900 nm), whose light cannot be perceived by these insects [23],

The behaviour of the insects in the 2×2 cm window where the heat source was presented to them in the first experiment was videotaped and subsequently analysed. The biting activity of each bug was analyzed during an interval of 10 min that was initiated after its first bite. In the second experiment, the analysis was carried out having the image of the 1×1 cm window projected on a TV screen. The location of the bites on this area was determined with 1 mm accuracy.

### Statistics

To test the precision of the bites performed by bugs on the linearly shaped heat source, we employed a quite conservative method. We measured the distance of each contact of the proboscis from the wire performed by each indivual along 10 min and computed a mean distance per insect. These means were compared between test (temperature of the wire different from the background) and control (same temperature) groups. Provided that data did not meet normality assumptions, we used the Mann-Whitney test. It should be noted that after some unsuccessful attempts to obtain food, the insects started to bite in zigzag (Fig. 2) along the wire, and therefore, we certainly underestimated the precision of the insects. However, as long as this would make test and control data more similar and not enhance differences, we decided to tolerate this bias. Despite this, differences revealed as highly significant.
